# μ-Acetato-aqua-μ-(5-bromo-2-{1,3-bis­[2-(5-bromo-2-oxidobenzyl­idene­amino)­eth­yl]imidazolidin-2-yl}phenolato)methano­ldinickel(II) methanol disolvate monohydrate

**DOI:** 10.1107/S1600536811035409

**Published:** 2011-09-14

**Authors:** Ahmed Raza Khan, Yohannes Tesema, Ray J. Butcher, Yilma Gultneh

**Affiliations:** a Department of Chemistry, Howard University, 525 College Street NW, Washington, DC 20059, USA

## Abstract

The crystal structure of the title compound, [Ni_2_(C_27_H_24_Br_3_N_4_O_3_)(CH_3_CO_2_)(CH_3_OH)(H_2_O)]·2CH_3_OH·H_2_O contains [*L*(OAc){(CH_3_OH)Ni}{(H_2_O)Ni}] mol­ecules {H_3_ *L* = 2-(5-bromo-2-hy­droxy­phen­yl)-1,3-bis­[4-(5-bromo-2-hy­droxy­phen­yl)-3-aza­but-3-en­yl]-1,3-imidazolidine} with additional water and two methanol solvent mol­ecules. In this instance, one of the two Ni atoms is coordinated to a water and the other to a methanol mol­ecule. The Ni—O and Ni—N distances, as well as the angles about the metal atoms, show quite regular octa­hedra around the central ions. The Ni—O_phenol_—Ni and Ni—O_acetate_—Ni angles are not similar [95.26 (13) and 97.34 (13)°, respectively], indicating that this subtle solvate exchange induces significant differences in the conformation adopted. The coordinated methanol ligand is involved in an intra­molecular hydrogen bond to the uncoordinated O atom of the bridging acetate ligand, while the coordinated water mol­ecule forms a hydrogen bond with the one of the methanol solvent mol­ecules. The water solvent mol­ecule forms strong hydrogen bonds to both phenolate O atoms. The remaining methanol solvent mol­ecule also forms a hydrogen bond with this solvent water mol­ecule.

## Related literature

For nickel complexes of similar ligands, see: Fondo *et al.* (2005 ▶, 2006**a* ▶,b* ▶, 2007 ▶, 2009 ▶); Khan *et al.* (2011 ▶); Lu *et al.* (2007 ▶); Paital *et al.* (2007 ▶, 2009 ▶).
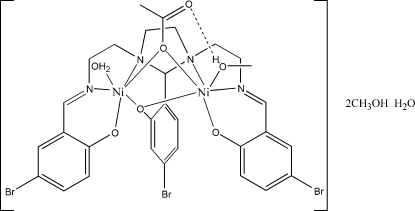


## Experimental

### 

#### Crystal data

[Ni_2_(C_27_H_24_Br_3_N_4_O_3_)(C_2_H_3_O_2_)(CH_4_O)(H_2_O)]·2CH_4_O·H_2_O
*M* *_r_* = 1000.85
Orthorhombic, 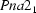

*a* = 14.7385 (16) Å
*b* = 18.552 (2) Å
*c* = 14.2504 (15) Å
*V* = 3896.4 (7) Å^3^
*Z* = 4
Mo *K*α radiation
μ = 4.10 mm^−1^
*T* = 168 K
0.49 × 0.12 × 0.06 mm

#### Data collection

Bruker SMART 1000 CCD diffractometer
Absorption correction: multi-scan (*SADABS*; Bruker, 2000 ▶) *T*_min_ = 0.676, *T*_max_ = 1.000
25239 measured reflections
8737 independent reflections
6627 reflections with *I* > 2σ(*I*)
*R*_int_ = 0.054

#### Refinement

*R*[*F* ^2^ > 2σ(*F* ^2^)] = 0.041
*wR*(*F* ^2^) = 0.092
*S* = 0.96
8737 reflections
479 parameters
7 restraints
H atoms treated by a mixture of independent and constrained refinement
Δρ_max_ = 0.71 e Å^−3^
Δρ_min_ = −0.73 e Å^−3^
Absolute structure: Flack (1983 ▶), 3686 Friedel pairs
Flack parameter: 0.007 (8)

### 

Data collection: *SMART* (Bruker, 2000 ▶); cell refinement: *SAINT-Plus* (Bruker, 2000 ▶); data reduction: *SAINT-Plus*; program(s) used to solve structure: *SHELXS97* (Sheldrick, 2008 ▶); program(s) used to refine structure: *SHELXL97* (Sheldrick, 2008 ▶); molecular graphics: *SHELXTL* (Sheldrick, 2008 ▶); software used to prepare material for publication: *SHELXTL*.

## Supplementary Material

Crystal structure: contains datablock(s) I, global. DOI: 10.1107/S1600536811035409/wm2523sup1.cif

Structure factors: contains datablock(s) I. DOI: 10.1107/S1600536811035409/wm2523Isup2.hkl

Additional supplementary materials:  crystallographic information; 3D view; checkCIF report

## Figures and Tables

**Table 1:** Hydrogen-bond geometry (Å, °)

*D*—H⋯*A*	*D*—H	H⋯*A*	*D*⋯*A*	*D*—H⋯*A*
O1*W*—H1*W*1⋯O2*W*^i^	0.80 (2)	2.01 (2)	2.810 (5)	174 (5)
O1*W*—H1*W*2⋯O3*M*	0.82 (2)	1.97 (3)	2.770 (5)	164 (5)
O2*W*—H2*W*1⋯O1*B*	0.80 (2)	1.86 (3)	2.631 (5)	160 (6)
O2*W*—H2*W*2⋯O1*A*	0.83 (2)	1.91 (2)	2.744 (5)	177 (6)
O1*M*—H1*M*⋯O2*AA*	0.84	1.77	2.602 (5)	174
O2*M*—H2*M*⋯O2*W*	0.84	1.89	2.725 (5)	172
O3*M*—H3*M*⋯O2*M*^i^	0.84	1.96	2.753 (6)	158

Symmetry code: (i) 
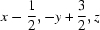
.

**Fig. 1.**
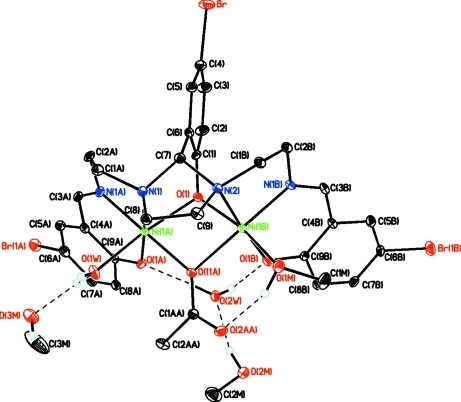
The molecular structure of compound (I), C32H43Br3N4Ni2O10, showing the atom labeling with displacement ellipsoids at the 30% probability level. All H atoms except those involved in the hydrogen bonding were removed for clarity. Hydrogen bonds are shown by dashed lines.

**Fig. 2.**
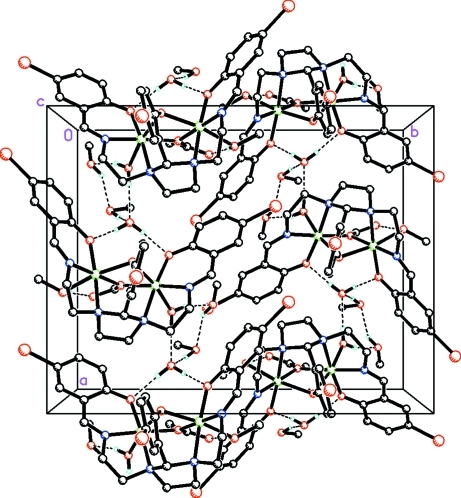
The molecular packing for C32H43Br3N4Ni2O10 viewed down the c axis. Hydrogen bonds are shown by dashed lines.

## supplementary crystallographic information

### Comment

Nickel complexes of the compartmental triprotic heptadentate ligand, 2-hydroxyphenyl-1,3-bis[4-(2-hydroxyphenyl)-3-azabut- 3-enyl]-1,3-imidazolidine and its derivatives have been of interest for their ability to give rise to dinuclear compounds with a predefined ground state (Fondo *et al.*, 2005, 2006**a*,b*, 2007, 2009; Lu *et al.*, 2007; Paital, *et al.*, 2007, 2009). Density functional theory (DFT) calculations demonstrated that the Schiff base provides an NCN bridge between the metal ions that helps to mediate the ferromagnetic exchange (Fondo, *et al.*, 2005). Consequently, the use of suitable cross-linking ligands between the dinuclear units could be a route to produce complexes of higher nuclearity, with all of the unpaired electrons aligned parallel to each other. The type of complex obtained depends on the synthesis conditions as the coordination environment about the metals is usually completed by coordinating solvent molecules.

The crystal structure shows that the title compound, [Ni_2_(CH_3_CO_2_)(C_27_H_24_Br_3_N_4_O_3_) (H_2_O)(CH_3_OH)]^.^2CH_3_OH^.^H_2_O, (I), contains [*L*(OAc){(CH_3_OH)Ni}{(H_2_O)Ni}] molecules (H_3_*L* = 2-(5-bromo-2-hydroxyphenyl)-1,3-bis[4-(5-bromo-2- hydroxyphenyl)-3-azabut-3-enyl]-1,3-imidazolidine) with water and two methanol molecules as solvates. In this instance one of the two nickel atoms is coordinated to a water and the other to a methanol molecule. This is in contrast to its related complex involving the ligand 2-(5-chloro-2-hydroxyphenyl)-1,3-bis[4-(5-chloro-2-hydroxyphenyl)-3-azabut-3- enyl]-1,3-imidazolidine, which was synthesized under similar conditions. In this case both nickel atoms contain coordinated methanol molecules (Khan *et al.*, 2011). It has previous been observed that nickel complexes involving this type of ligand are prone to solvate exchange (Fondo *et al.*, 2009).

(I) is a neutral dinuclear compound, where the *L*^3-^ Schiff base acts as a trianionic heptadentate ligand, using each one of its N_2_O compartments to coordinate a nickel atom. Thus, the metal atoms are joined to one terminal phenol oxygen (O1A, O1B), an iminic nitrogen (N1A, N1B), and an aminic nitrogen atom (N1, N2), with the aminic NCN group (N2—C7—N2) acting as a bridge between both nickel ions. In addition, the nickel atoms are linked by the endogenous phenolate oxygen atom (O1) of the central ligand arm and by an exogenous bridging monodentate acetate group (O11A). This gives rise to a nearly planar Ni_2_O_2_ metallacycle, with an intramolecular Ni—Ni distance of 3.0927 (9) Å. The coordination spheres of the nickel atoms are completed by solvent molecules. In the case of Ni1A by water and in the case of Ni1B by methanol molecules. Therefore, the metal atoms are hexacoordinated in a N_2_O_4_ environment, with an octahedral geometry. The Ni—O and Ni—N distances, as well as the angles about the metal atoms, show quite regular polyhedra around the central ions. However, unlike the analogous complex formed with 2-(5-chloro-2-hydroxyphenyl)-1,3-bis[4-(5-chloro-2- hydroxyphenyl)-3-azabut-3-enyl]-1,3-imidazolidine (Khan *et al.*, 2011) the Ni—O_phenol_—Ni and Ni—O_acetate_—Ni angles are not similar [95.26 (13)° and 97.34 (13)°, respectively] and more closely related to a similar complex (Fondo *et al.*, 2009) with a similar coordination environment about the two Ni atoms (one with water and the other with methanol coordinated). Thus this subtle solvate exchange induces significant differences in the conformation adopted. There are structures of Ni complexes involving similar ligands reported in the literature which differ only in the nature of the coordinating solvent (H_2_O) and solvate molecules (H_2_O, CH_3_CN) in the lattice (Fondo *et al.*, 2006*b*) and similar differences are observed.

The coordinated methanol ligand is involved in an intramolecular hydrogen bond to the uncoordinated O atom (O2AA) of the bridging acetate ligand while the coordinated water molecule forms a hydrogen bond with the one of the methanol solvate molecules. The solvate water molecule forms strong hydrogen bonds to both O1A and O1B. The remaining methanol solvate molecule also forms a hydrogen bond with this water solvate molecule.

### Experimental

For the synthesis of the ligand (H_3_*L*) methanol solutions of triethylenetetramine and 5-bromosalicylaldehyde were mixed in a 1:3 molar ratio. After heating at 333 K for a few minutes, diethylether was added to this mixture, and yellow crystals were separated, filtered and recrystallized from methanol solution: Mp 376 K. For synthesis of the complex, to a stirred methanol solution (25 ml) of [Ni(OAc)_2_]^.^4H_2_O (1.5 g, 2.67 mmol) was added 1.33 g (5.35 mmol) of the ligand H_3_*L*. Slow evaporation of the green filtrate overnight yielded green to brownish cystal suitable for X-ray analysis in 70% yield.

### Refinement

H atoms were placed in geometrically idealized positions and constrained to ride on their parent atoms with an O—H distance of 0.84 and C—H distances of 0.95 - 0.99 Å [*U*_iso_(H) = 1.2*U*_eq_(OH, CH, CH_2_) [*U*_iso_(H) = 1.5*U*_eq_(CH_3_)]. Water H atoms were refined isotropically with O—H distances restrained to 0.82 Å and H—O—H angle to 104.5° with [*U*_iso_(H) = 1.5*U*_eq_(O)].

### Crystal data

**Table:**

[Ni_2_(C_27_H_24_Br_3_N_4_O_3_)(C_2_H_3_O_2_)(CH_4_O)(H_2_O)]·2CH_4_O·H_2_O	*F*(000) = 2016
*M**_r_* = 1000.85	*D*_x_ = 1.706 Mg m^−^^3^
Orthorhombic, *P**n**a*2_1_	Mo *K*α radiation, λ = 0.71073 Å
Hall symbol: P 2c -2n	Cell parameters from 7492 reflections
*a* = 14.7385 (16) Å	θ = 2.3–26.7°
*b* = 18.552 (2) Å	µ = 4.10 mm^−^^1^
*c* = 14.2504 (15) Å	*T* = 168 K
*V* = 3896.4 (7) Å^3^	Needle, brown
*Z* = 4	0.49 × 0.12 × 0.06 mm

### Data collection

**Table:**

Bruker SMART 1000 CCD diffractometer	8737 independent reflections
Radiation source: fine-focus sealed tube	6627 reflections with *I* > 2σ(*I*)
graphite	*R*_int_ = 0.054
φ and ω scans	θ_max_ = 28.3°, θ_min_ = 1.8°
Absorption correction: multi-scan (*SADABS*; Bruker, 2000)	*h* = −19→16
*T*_min_ = 0.676, *T*_max_ = 1.000	*k* = −24→19
25239 measured reflections	*l* = −18→14

### Refinement

**Table:**

Refinement on *F*^2^	Secondary atom site location: difference Fourier map
Least-squares matrix: full	Hydrogen site location: inferred from neighbouring sites
*R*[*F*^2^ > 2σ(*F*^2^)] = 0.041	H atoms treated by a mixture of independent and constrained refinement
*wR*(*F*^2^) = 0.092	*w* = 1/[σ^2^(*F*_o_^2^) + (0.0457*P*)^2^] where *P* = (*F*_o_^2^ + 2*F*_c_^2^)/3
*S* = 0.96	(Δ/σ)_max_ = 0.001
8737 reflections	Δρ_max_ = 0.71 e Å^−^^3^
479 parameters	Δρ_min_ = −0.73 e Å^−^^3^
7 restraints	Absolute structure: Flack (1983), 3686 Friedel pairs
Primary atom site location: structure-invariant direct methods	Flack parameter: 0.007 (8)

### Special details

**Table:**

Geometry. All e.s.d.'s (except the e.s.d. in the dihedral angle between two l.s. planes) are estimated using the full covariance matrix. The cell e.s.d.'s are taken into account individually in the estimation of e.s.d.'s in distances, angles and torsion angles; correlations between e.s.d.'s in cell parameters are only used when they are defined by crystal symmetry. An approximate (isotropic) treatment of cell e.s.d.'s is used for estimating e.s.d.'s involving l.s. planes.
Refinement. Refinement of *F*^2^ against ALL reflections. The weighted *R*-factor *wR* and goodness of fit *S* are based on *F*^2^, conventional *R*-factors *R* are based on *F*, with *F* set to zero for negative *F*^2^. The threshold expression of *F*^2^ > σ(*F*^2^) is used only for calculating *R*-factors(gt) *etc*. and is not relevant to the choice of reflections for refinement. *R*-factors based on *F*^2^ are statistically about twice as large as those based on *F*, and *R*- factors based on ALL data will be even larger.

### Fractional atomic coordinates and isotropic or equivalent isotropic displacement parameters (Å^2^)

**Table:**

	*x*	*y*	*z*	*U*_iso_*/*U*_eq_	
Ni1A	0.91124 (4)	0.76904 (3)	0.22387 (4)	0.02018 (13)	
Ni1B	0.94953 (4)	0.60931 (3)	0.27176 (4)	0.02102 (13)	
Br	0.94521 (4)	0.74287 (4)	0.73692 (4)	0.04501 (16)	
Br1A	1.18571 (4)	1.09747 (3)	0.11852 (4)	0.04143 (15)	
Br1B	1.35626 (4)	0.37081 (3)	0.29322 (5)	0.04296 (16)	
O1	0.9917 (2)	0.71201 (17)	0.3177 (2)	0.0215 (7)	
O1A	1.0148 (2)	0.80326 (17)	0.1449 (2)	0.0242 (7)	
O1B	1.0661 (2)	0.58968 (18)	0.2059 (2)	0.0270 (8)	
O11A	0.9104 (2)	0.67067 (18)	0.1578 (2)	0.0223 (7)	
O2AA	0.8697 (3)	0.5877 (2)	0.0550 (3)	0.0340 (9)	
O1W	0.8185 (2)	0.8101 (2)	0.1226 (3)	0.0331 (8)	
H1W1	0.7642 (13)	0.808 (3)	0.119 (4)	0.050*	
H1W2	0.835 (3)	0.844 (2)	0.089 (4)	0.050*	
O2W	1.1295 (2)	0.69264 (18)	0.0972 (2)	0.0261 (8)	
H2W1	1.106 (3)	0.6574 (16)	0.119 (4)	0.039*	
H2W2	1.096 (3)	0.7270 (17)	0.113 (4)	0.039*	
O1M	0.8879 (2)	0.51575 (18)	0.2106 (3)	0.0320 (9)	
H1M	0.8806	0.5361	0.1585	0.038*	
O2M	1.1750 (3)	0.6277 (2)	−0.0678 (3)	0.0433 (10)	
H2M	1.1557	0.6474	−0.0185	0.052*	
O3M	0.8398 (3)	0.9221 (3)	−0.0041 (3)	0.0471 (11)	
H3M	0.7981	0.8972	−0.0278	0.057*	
N1	0.7953 (2)	0.7411 (2)	0.3129 (3)	0.0208 (9)	
N2	0.8242 (3)	0.6225 (2)	0.3479 (3)	0.0212 (9)	
N1A	0.9061 (2)	0.8629 (2)	0.2906 (3)	0.0227 (9)	
N1B	0.9873 (3)	0.5495 (2)	0.3811 (3)	0.0237 (9)	
C1	0.9803 (3)	0.7248 (3)	0.4090 (3)	0.0230 (11)	
C2	1.0506 (3)	0.7471 (3)	0.4670 (4)	0.0336 (13)	
H2A	1.1075	0.7582	0.4392	0.040*	
C3	1.0418 (4)	0.7540 (3)	0.5630 (4)	0.0380 (14)	
H3A	1.0920	0.7689	0.6001	0.046*	
C4	0.9591 (4)	0.7391 (3)	0.6046 (4)	0.0309 (12)	
C5	0.8851 (3)	0.7210 (3)	0.5493 (3)	0.0255 (11)	
H5A	0.8275	0.7132	0.5775	0.031*	
C6	0.8950 (3)	0.7144 (3)	0.4532 (3)	0.0219 (11)	
C7	0.8156 (3)	0.6939 (3)	0.3951 (3)	0.0233 (11)	
H7A	0.7608	0.6927	0.4364	0.028*	
C8	0.7254 (3)	0.6988 (3)	0.2607 (3)	0.0239 (11)	
H8A	0.7336	0.7040	0.1921	0.029*	
H8B	0.6635	0.7149	0.2777	0.029*	
C9	0.7410 (3)	0.6207 (3)	0.2912 (4)	0.0267 (11)	
H9A	0.6892	0.6029	0.3288	0.032*	
H9B	0.7489	0.5891	0.2359	0.032*	
C1A	0.7612 (4)	0.8134 (3)	0.3400 (4)	0.0293 (12)	
H1AA	0.7203	0.8085	0.3947	0.035*	
H1AB	0.7253	0.8337	0.2874	0.035*	
C2A	0.8367 (4)	0.8651 (3)	0.3643 (4)	0.0286 (12)	
H2AA	0.8123	0.9146	0.3700	0.034*	
H2AB	0.8640	0.8515	0.4253	0.034*	
C3A	0.9487 (3)	0.9210 (2)	0.2692 (4)	0.0243 (10)	
H3AA	0.9335	0.9634	0.3031	0.029*	
C4A	1.0182 (3)	0.9272 (3)	0.1972 (3)	0.0235 (11)	
C5A	1.0590 (3)	0.9946 (3)	0.1885 (3)	0.0276 (12)	
H5AA	1.0396	1.0331	0.2275	0.033*	
C6A	1.1270 (3)	1.0062 (3)	0.1243 (4)	0.0311 (12)	
C7A	1.1550 (4)	0.9513 (3)	0.0658 (4)	0.0302 (12)	
H7AA	1.2014	0.9597	0.0208	0.036*	
C8A	1.1158 (3)	0.8849 (3)	0.0731 (4)	0.0259 (11)	
H8AA	1.1348	0.8478	0.0315	0.031*	
C9A	1.0476 (3)	0.8692 (3)	0.1405 (3)	0.0211 (10)	
C1B	0.8263 (3)	0.5603 (3)	0.4150 (3)	0.0266 (12)	
H1BA	0.8070	0.5161	0.3816	0.032*	
H1BB	0.7823	0.5693	0.4661	0.032*	
C2B	0.9192 (3)	0.5476 (3)	0.4575 (3)	0.0268 (12)	
H2BA	0.9328	0.5855	0.5044	0.032*	
H2BB	0.9208	0.5003	0.4894	0.032*	
C3B	1.0569 (3)	0.5090 (3)	0.3867 (4)	0.0258 (11)	
H3BA	1.0640	0.4810	0.4421	0.031*	
C4B	1.1270 (3)	0.5023 (3)	0.3140 (3)	0.0220 (10)	
C5B	1.1949 (3)	0.4516 (3)	0.3329 (4)	0.0284 (12)	
H5BA	1.1935	0.4248	0.3897	0.034*	
C6B	1.2640 (3)	0.4404 (2)	0.2688 (4)	0.0255 (11)	
C7B	1.2655 (3)	0.4783 (3)	0.1860 (4)	0.0267 (11)	
H7BA	1.3128	0.4701	0.1420	0.032*	
C8B	1.1996 (3)	0.5278 (3)	0.1668 (4)	0.0254 (11)	
H8BA	1.2023	0.5538	0.1095	0.030*	
C9B	1.1275 (3)	0.5415 (2)	0.2293 (4)	0.0221 (10)	
C1AA	0.8968 (3)	0.6495 (3)	0.0734 (4)	0.0246 (11)	
C2AA	0.9144 (4)	0.7004 (3)	−0.0069 (4)	0.0354 (14)	
H2AC	0.9538	0.6769	−0.0531	0.053*	
H2AD	0.9441	0.7440	0.0168	0.053*	
H2AE	0.8567	0.7134	−0.0366	0.053*	
C1M	0.9324 (4)	0.4483 (3)	0.1970 (5)	0.0475 (17)	
H1MA	0.8951	0.4175	0.1565	0.071*	
H1MB	0.9411	0.4246	0.2578	0.071*	
H1MC	0.9915	0.4564	0.1673	0.071*	
C2M	1.1886 (5)	0.6806 (4)	−0.1371 (5)	0.067 (2)	
H2MA	1.2129	0.6579	−0.1939	0.101*	
H2MB	1.2317	0.7167	−0.1141	0.101*	
H2MC	1.1306	0.7039	−0.1517	0.101*	
C3M	0.9083 (6)	0.9318 (7)	−0.0695 (6)	0.119 (5)	
H3M1	0.9358	0.8851	−0.0844	0.178*	
H3M2	0.9546	0.9640	−0.0436	0.178*	
H3M3	0.8829	0.9531	−0.1267	0.178*	

### Atomic displacement parameters (Å^2^)

**Table:**

	*U*^11^	*U*^22^	*U*^33^	*U*^12^	*U*^13^	*U*^23^
Ni1A	0.0220 (3)	0.0193 (3)	0.0192 (3)	0.0021 (2)	0.0017 (3)	0.0004 (3)
Ni1B	0.0247 (3)	0.0203 (3)	0.0181 (3)	0.0015 (2)	0.0013 (3)	0.0023 (3)
Br	0.0482 (3)	0.0678 (4)	0.0190 (3)	0.0032 (3)	−0.0009 (3)	−0.0048 (3)
Br1A	0.0510 (4)	0.0299 (3)	0.0434 (3)	−0.0129 (3)	−0.0024 (3)	0.0073 (3)
Br1B	0.0395 (3)	0.0385 (3)	0.0509 (4)	0.0175 (2)	0.0036 (3)	0.0121 (3)
O1	0.0211 (17)	0.0257 (18)	0.0178 (17)	0.0023 (13)	0.0012 (13)	0.0022 (14)
O1A	0.0263 (18)	0.0211 (19)	0.0253 (19)	0.0010 (14)	0.0037 (14)	−0.0027 (15)
O1B	0.0300 (19)	0.0273 (19)	0.0239 (19)	0.0065 (14)	0.0047 (14)	0.0101 (15)
O11A	0.0271 (18)	0.0228 (19)	0.0169 (17)	0.0012 (14)	−0.0042 (14)	0.0006 (14)
O2AA	0.046 (2)	0.031 (2)	0.025 (2)	−0.0039 (17)	−0.0010 (17)	−0.0087 (17)
O1W	0.0222 (17)	0.036 (2)	0.041 (2)	0.0025 (17)	−0.0027 (17)	0.016 (2)
O2W	0.0271 (19)	0.0209 (19)	0.030 (2)	0.0018 (15)	0.0038 (15)	0.0039 (16)
O1M	0.038 (2)	0.026 (2)	0.032 (2)	−0.0018 (16)	−0.0003 (17)	0.0011 (17)
O2M	0.065 (3)	0.034 (2)	0.032 (2)	0.007 (2)	0.003 (2)	−0.0002 (19)
O3M	0.043 (3)	0.057 (3)	0.042 (3)	−0.008 (2)	0.0026 (19)	0.019 (2)
N1	0.0168 (19)	0.025 (2)	0.021 (2)	0.0044 (16)	0.0005 (15)	0.0000 (18)
N2	0.026 (2)	0.020 (2)	0.018 (2)	−0.0001 (17)	−0.0020 (16)	0.0008 (17)
N1A	0.0210 (19)	0.020 (2)	0.027 (2)	0.0050 (16)	0.0055 (19)	0.0004 (18)
N1B	0.030 (2)	0.026 (2)	0.015 (2)	0.0004 (18)	−0.0009 (17)	0.0049 (18)
C1	0.025 (3)	0.023 (3)	0.020 (2)	0.007 (2)	0.000 (2)	−0.005 (2)
C2	0.023 (3)	0.050 (4)	0.028 (3)	0.002 (3)	−0.001 (2)	−0.010 (3)
C3	0.031 (3)	0.056 (4)	0.027 (3)	0.003 (3)	−0.008 (2)	−0.011 (3)
C4	0.035 (3)	0.036 (3)	0.021 (3)	0.008 (2)	−0.001 (2)	−0.001 (2)
C5	0.025 (3)	0.030 (3)	0.021 (3)	0.001 (2)	0.003 (2)	0.000 (2)
C6	0.024 (3)	0.022 (3)	0.019 (2)	0.005 (2)	−0.002 (2)	0.000 (2)
C7	0.023 (3)	0.025 (3)	0.023 (3)	−0.001 (2)	0.004 (2)	0.000 (2)
C8	0.023 (2)	0.026 (3)	0.023 (3)	0.0025 (19)	0.0003 (19)	0.001 (2)
C9	0.024 (2)	0.029 (3)	0.027 (3)	−0.006 (2)	−0.001 (2)	0.003 (2)
C1A	0.032 (3)	0.030 (3)	0.026 (3)	0.003 (2)	0.003 (2)	0.001 (2)
C2A	0.036 (3)	0.023 (3)	0.027 (3)	0.006 (2)	0.014 (2)	−0.002 (2)
C3A	0.029 (2)	0.017 (2)	0.027 (3)	0.002 (2)	−0.001 (2)	−0.006 (2)
C4A	0.026 (2)	0.021 (3)	0.024 (3)	−0.001 (2)	−0.002 (2)	0.005 (2)
C5A	0.029 (3)	0.029 (3)	0.025 (3)	0.002 (2)	−0.002 (2)	−0.001 (2)
C6A	0.031 (3)	0.029 (3)	0.034 (3)	−0.003 (2)	−0.004 (2)	0.007 (3)
C7A	0.032 (3)	0.031 (3)	0.028 (3)	0.002 (2)	0.004 (2)	0.010 (2)
C8A	0.029 (3)	0.025 (3)	0.025 (3)	0.004 (2)	0.002 (2)	0.005 (2)
C9A	0.021 (2)	0.022 (3)	0.021 (2)	0.0049 (19)	−0.0039 (19)	0.003 (2)
C1B	0.030 (3)	0.028 (3)	0.021 (3)	0.002 (2)	0.009 (2)	0.008 (2)
C2B	0.031 (3)	0.027 (3)	0.022 (3)	0.003 (2)	0.010 (2)	0.008 (2)
C3B	0.029 (3)	0.024 (3)	0.024 (3)	0.002 (2)	−0.003 (2)	0.009 (2)
C4B	0.022 (2)	0.022 (3)	0.021 (3)	−0.0018 (19)	−0.0024 (19)	0.007 (2)
C5B	0.032 (3)	0.021 (3)	0.032 (3)	−0.001 (2)	−0.002 (2)	0.005 (2)
C6B	0.027 (3)	0.019 (2)	0.031 (3)	0.0052 (19)	−0.001 (2)	0.004 (2)
C7B	0.027 (3)	0.024 (3)	0.029 (3)	0.006 (2)	0.007 (2)	−0.004 (2)
C8B	0.029 (3)	0.023 (3)	0.024 (3)	0.002 (2)	0.003 (2)	0.004 (2)
C9B	0.028 (2)	0.018 (2)	0.021 (2)	0.0002 (19)	0.000 (2)	0.000 (2)
C1AA	0.023 (3)	0.028 (3)	0.022 (3)	0.004 (2)	0.000 (2)	−0.001 (2)
C2AA	0.040 (3)	0.043 (4)	0.023 (3)	−0.005 (3)	−0.005 (2)	0.006 (3)
C1M	0.049 (4)	0.029 (3)	0.064 (5)	−0.001 (3)	−0.007 (3)	−0.009 (3)
C2M	0.082 (6)	0.065 (5)	0.055 (5)	−0.010 (4)	−0.001 (4)	0.014 (4)
C3M	0.048 (5)	0.231 (13)	0.077 (7)	0.013 (7)	0.019 (4)	0.088 (8)

### Geometric parameters (Å, °)

**Table:**

Ni1A—N1A	1.986 (4)	C7—H7A	1.0000
Ni1A—O1A	2.000 (3)	C8—C9	1.529 (7)
Ni1A—O11A	2.053 (3)	C8—H8A	0.9900
Ni1A—O1	2.077 (3)	C8—H8B	0.9900
Ni1A—O1W	2.129 (3)	C9—H9A	0.9900
Ni1A—N1	2.190 (4)	C9—H9B	0.9900
Ni1A—Ni1B	3.0927 (9)	C1A—C2A	1.510 (7)
Ni1B—O1B	1.991 (3)	C1A—H1AA	0.9900
Ni1B—N1B	1.992 (4)	C1A—H1AB	0.9900
Ni1B—O11A	2.065 (3)	C2A—H2AA	0.9900
Ni1B—O1	2.109 (3)	C2A—H2AB	0.9900
Ni1B—O1M	2.144 (3)	C3A—C4A	1.455 (7)
Ni1B—N2	2.157 (4)	C3A—H3AA	0.9500
Br—C4	1.898 (5)	C4A—C5A	1.392 (7)
Br1A—C6A	1.904 (5)	C4A—C9A	1.413 (7)
Br1B—C6B	1.907 (5)	C5A—C6A	1.373 (7)
O1—C1	1.333 (5)	C5A—H5AA	0.9500
O1A—C9A	1.317 (6)	C6A—C7A	1.378 (8)
O1B—C9B	1.315 (5)	C7A—C8A	1.365 (7)
O11A—C1AA	1.282 (6)	C7A—H7AA	0.9500
O2AA—C1AA	1.243 (6)	C8A—C9A	1.421 (7)
O1W—H1W1	0.802 (19)	C8A—H8AA	0.9500
O1W—H1W2	0.823 (19)	C1B—C2B	1.515 (7)
O2W—H2W1	0.803 (19)	C1B—H1BA	0.9900
O2W—H2W2	0.832 (19)	C1B—H1BB	0.9900
O1M—C1M	1.426 (6)	C2B—H2BA	0.9900
O1M—H1M	0.8400	C2B—H2BB	0.9900
O2M—C2M	1.406 (8)	C3B—C4B	1.469 (7)
O2M—H2M	0.8400	C3B—H3BA	0.9500
O3M—C3M	1.385 (8)	C4B—C5B	1.400 (7)
O3M—H3M	0.8400	C4B—C9B	1.409 (6)
N1—C1A	1.485 (6)	C5B—C6B	1.383 (7)
N1—C7	1.493 (6)	C5B—H5BA	0.9500
N1—C8	1.493 (6)	C6B—C7B	1.374 (7)
N2—C9	1.469 (6)	C7B—C8B	1.365 (7)
N2—C7	1.490 (6)	C7B—H7BA	0.9500
N2—C1B	1.499 (6)	C8B—C9B	1.409 (7)
N1A—C3A	1.284 (6)	C8B—H8BA	0.9500
N1A—C2A	1.466 (6)	C1AA—C2AA	1.505 (7)
N1B—C3B	1.274 (6)	C2AA—H2AC	0.9800
N1B—C2B	1.481 (6)	C2AA—H2AD	0.9800
C1—C2	1.388 (7)	C2AA—H2AE	0.9800
C1—C6	1.419 (7)	C1M—H1MA	0.9800
C2—C3	1.380 (7)	C1M—H1MB	0.9800
C2—H2A	0.9500	C1M—H1MC	0.9800
C3—C4	1.383 (8)	C2M—H2MA	0.9800
C3—H3A	0.9500	C2M—H2MB	0.9800
C4—C5	1.387 (7)	C2M—H2MC	0.9800
C5—C6	1.383 (7)	C3M—H3M1	0.9800
C5—H5A	0.9500	C3M—H3M2	0.9800
C6—C7	1.484 (7)	C3M—H3M3	0.9800
			
N1A—Ni1A—O1A	91.15 (14)	C9—C8—H8A	110.8
N1A—Ni1A—O11A	177.16 (14)	N1—C8—H8B	110.8
O1A—Ni1A—O11A	91.63 (13)	C9—C8—H8B	110.8
N1A—Ni1A—O1	99.20 (15)	H8A—C8—H8B	108.9
O1A—Ni1A—O1	95.07 (13)	N2—C9—C8	105.1 (4)
O11A—Ni1A—O1	81.13 (13)	N2—C9—H9A	110.7
N1A—Ni1A—O1W	89.21 (16)	C8—C9—H9A	110.7
O1A—Ni1A—O1W	89.69 (14)	N2—C9—H9B	110.7
O11A—Ni1A—O1W	90.22 (14)	C8—C9—H9B	110.7
O1—Ni1A—O1W	170.23 (14)	H9A—C9—H9B	108.8
N1A—Ni1A—N1	84.31 (15)	N1—C1A—C2A	112.6 (4)
O1A—Ni1A—N1	175.19 (14)	N1—C1A—H1AA	109.1
O11A—Ni1A—N1	92.89 (14)	C2A—C1A—H1AA	109.1
O1—Ni1A—N1	87.26 (13)	N1—C1A—H1AB	109.1
O1W—Ni1A—N1	88.65 (14)	C2A—C1A—H1AB	109.1
N1A—Ni1A—Ni1B	137.86 (12)	H1AA—C1A—H1AB	107.8
O1A—Ni1A—Ni1B	106.81 (9)	N1A—C2A—C1A	109.4 (4)
O11A—Ni1A—Ni1B	41.47 (9)	N1A—C2A—H2AA	109.8
O1—Ni1A—Ni1B	42.76 (9)	C1A—C2A—H2AA	109.8
O1W—Ni1A—Ni1B	127.59 (11)	N1A—C2A—H2AB	109.8
N1—Ni1A—Ni1B	77.74 (10)	C1A—C2A—H2AB	109.8
O1B—Ni1B—N1B	91.46 (15)	H2AA—C2A—H2AB	108.2
O1B—Ni1B—O11A	88.35 (13)	N1A—C3A—C4A	125.3 (4)
N1B—Ni1B—O11A	179.59 (16)	N1A—C3A—H3AA	117.3
O1B—Ni1B—O1	93.27 (14)	C4A—C3A—H3AA	117.3
N1B—Ni1B—O1	100.24 (15)	C5A—C4A—C9A	120.0 (4)
O11A—Ni1B—O1	80.13 (13)	C5A—C4A—C3A	116.0 (4)
O1B—Ni1B—O1M	91.50 (14)	C9A—C4A—C3A	123.9 (4)
N1B—Ni1B—O1M	89.16 (16)	C6A—C5A—C4A	121.0 (5)
O11A—Ni1B—O1M	90.49 (13)	C6A—C5A—H5AA	119.5
O1—Ni1B—O1M	169.33 (13)	C4A—C5A—H5AA	119.5
O1B—Ni1B—N2	175.60 (14)	C5A—C6A—C7A	120.4 (5)
N1B—Ni1B—N2	84.77 (16)	C5A—C6A—Br1A	120.0 (4)
O11A—Ni1B—N2	95.40 (14)	C7A—C6A—Br1A	119.6 (4)
O1—Ni1B—N2	89.65 (13)	C8A—C7A—C6A	119.6 (5)
O1M—Ni1B—N2	86.18 (14)	C8A—C7A—H7AA	120.2
O1B—Ni1B—Ni1A	103.23 (10)	C6A—C7A—H7AA	120.2
N1B—Ni1B—Ni1A	139.22 (12)	C7A—C8A—C9A	122.4 (5)
O11A—Ni1B—Ni1A	41.19 (9)	C7A—C8A—H8AA	118.8
O1—Ni1B—Ni1A	41.98 (8)	C9A—C8A—H8AA	118.8
O1M—Ni1B—Ni1A	127.49 (10)	O1A—C9A—C4A	124.6 (4)
N2—Ni1B—Ni1A	81.14 (11)	O1A—C9A—C8A	118.8 (4)
C1—O1—Ni1A	117.8 (3)	C4A—C9A—C8A	116.6 (4)
C1—O1—Ni1B	115.3 (3)	N2—C1B—C2B	113.2 (4)
Ni1A—O1—Ni1B	95.26 (13)	N2—C1B—H1BA	108.9
C9A—O1A—Ni1A	127.0 (3)	C2B—C1B—H1BA	108.9
C9B—O1B—Ni1B	126.8 (3)	N2—C1B—H1BB	108.9
C1AA—O11A—Ni1A	134.7 (3)	C2B—C1B—H1BB	108.9
C1AA—O11A—Ni1B	127.8 (3)	H1BA—C1B—H1BB	107.8
Ni1A—O11A—Ni1B	97.34 (13)	N1B—C2B—C1B	108.3 (4)
Ni1A—O1W—H1W1	132 (4)	N1B—C2B—H2BA	110.0
Ni1A—O1W—H1W2	119 (4)	C1B—C2B—H2BA	110.0
H1W1—O1W—H1W2	107 (3)	N1B—C2B—H2BB	110.0
H2W1—O2W—H2W2	105 (3)	C1B—C2B—H2BB	110.0
C1M—O1M—Ni1B	124.8 (3)	H2BA—C2B—H2BB	108.4
C1M—O1M—H1M	109.5	N1B—C3B—C4B	124.9 (5)
Ni1B—O1M—H1M	92.8	N1B—C3B—H3BA	117.6
C2M—O2M—H2M	109.5	C4B—C3B—H3BA	117.6
C3M—O3M—H3M	109.5	C5B—C4B—C9B	120.5 (4)
C1A—N1—C7	113.2 (4)	C5B—C4B—C3B	115.0 (4)
C1A—N1—C8	111.8 (4)	C9B—C4B—C3B	124.4 (4)
C7—N1—C8	102.7 (4)	C6B—C5B—C4B	120.0 (5)
C1A—N1—Ni1A	101.6 (3)	C6B—C5B—H5BA	120.0
C7—N1—Ni1A	115.9 (3)	C4B—C5B—H5BA	120.0
C8—N1—Ni1A	112.0 (3)	C7B—C6B—C5B	120.1 (4)
C9—N2—C7	101.4 (4)	C7B—C6B—Br1B	119.4 (4)
C9—N2—C1B	110.6 (4)	C5B—C6B—Br1B	120.4 (4)
C7—N2—C1B	113.5 (4)	C8B—C7B—C6B	120.3 (5)
C9—N2—Ni1B	115.8 (3)	C8B—C7B—H7BA	119.8
C7—N2—Ni1B	113.6 (3)	C6B—C7B—H7BA	119.8
C1B—N2—Ni1B	102.4 (3)	C7B—C8B—C9B	122.1 (5)
C3A—N1A—C2A	119.2 (4)	C7B—C8B—H8BA	119.0
C3A—N1A—Ni1A	127.1 (3)	C9B—C8B—H8BA	119.0
C2A—N1A—Ni1A	113.2 (3)	O1B—C9B—C4B	124.3 (4)
C3B—N1B—C2B	119.1 (4)	O1B—C9B—C8B	118.8 (4)
C3B—N1B—Ni1B	127.1 (3)	C4B—C9B—C8B	116.9 (4)
C2B—N1B—Ni1B	113.5 (3)	O2AA—C1AA—O11A	122.1 (5)
O1—C1—C2	122.7 (5)	O2AA—C1AA—C2AA	118.3 (5)
O1—C1—C6	121.4 (4)	O11A—C1AA—C2AA	119.6 (5)
C2—C1—C6	115.9 (5)	C1AA—C2AA—H2AC	109.5
C3—C2—C1	123.2 (5)	C1AA—C2AA—H2AD	109.5
C3—C2—H2A	118.4	H2AC—C2AA—H2AD	109.5
C1—C2—H2A	118.4	C1AA—C2AA—H2AE	109.5
C2—C3—C4	119.3 (5)	H2AC—C2AA—H2AE	109.5
C2—C3—H3A	120.4	H2AD—C2AA—H2AE	109.5
C4—C3—H3A	120.4	O1M—C1M—H1MA	109.5
C3—C4—C5	119.9 (5)	O1M—C1M—H1MB	109.5
C3—C4—Br	120.9 (4)	H1MA—C1M—H1MB	109.5
C5—C4—Br	119.2 (4)	O1M—C1M—H1MC	109.5
C6—C5—C4	120.0 (5)	H1MA—C1M—H1MC	109.5
C6—C5—H5A	120.0	H1MB—C1M—H1MC	109.5
C4—C5—H5A	120.0	O2M—C2M—H2MA	109.5
C5—C6—C1	121.4 (5)	O2M—C2M—H2MB	109.5
C5—C6—C7	119.5 (4)	H2MA—C2M—H2MB	109.5
C1—C6—C7	119.1 (4)	O2M—C2M—H2MC	109.5
C6—C7—N2	114.4 (4)	H2MA—C2M—H2MC	109.5
C6—C7—N1	116.5 (4)	H2MB—C2M—H2MC	109.5
N2—C7—N1	100.6 (4)	O3M—C3M—H3M1	109.5
C6—C7—H7A	108.3	O3M—C3M—H3M2	109.5
N2—C7—H7A	108.3	H3M1—C3M—H3M2	109.5
N1—C7—H7A	108.3	O3M—C3M—H3M3	109.5
N1—C8—C9	104.6 (4)	H3M1—C3M—H3M3	109.5
N1—C8—H8A	110.8	H3M2—C3M—H3M3	109.5
			
N1A—Ni1A—Ni1B—O1B	−112.42 (19)	O1M—Ni1B—N2—C1B	69.5 (3)
O1A—Ni1A—Ni1B—O1B	−1.01 (14)	Ni1A—Ni1B—N2—C1B	−161.6 (3)
O11A—Ni1A—Ni1B—O1B	71.72 (18)	O1A—Ni1A—N1A—C3A	9.8 (4)
O1—Ni1A—Ni1B—O1B	−80.01 (16)	O1—Ni1A—N1A—C3A	105.1 (4)
O1W—Ni1A—Ni1B—O1B	102.02 (17)	O1W—Ni1A—N1A—C3A	−79.9 (4)
N1—Ni1A—Ni1B—O1B	−179.38 (15)	N1—Ni1A—N1A—C3A	−168.6 (4)
N1A—Ni1A—Ni1B—N1B	−4.2 (2)	Ni1B—Ni1A—N1A—C3A	126.8 (4)
O1A—Ni1A—Ni1B—N1B	107.2 (2)	O1A—Ni1A—N1A—C2A	−178.3 (3)
O11A—Ni1A—Ni1B—N1B	179.9 (2)	O1—Ni1A—N1A—C2A	−82.9 (3)
O1—Ni1A—Ni1B—N1B	28.2 (2)	O1W—Ni1A—N1A—C2A	92.1 (3)
O1W—Ni1A—Ni1B—N1B	−149.8 (2)	N1—Ni1A—N1A—C2A	3.3 (3)
N1—Ni1A—Ni1B—N1B	−71.2 (2)	Ni1B—Ni1A—N1A—C2A	−61.3 (4)
N1A—Ni1A—Ni1B—O11A	175.9 (2)	O1B—Ni1B—N1B—C3B	−8.8 (5)
O1A—Ni1A—Ni1B—O11A	−72.73 (17)	O1—Ni1B—N1B—C3B	−102.4 (4)
O1—Ni1A—Ni1B—O11A	−151.73 (19)	O1M—Ni1B—N1B—C3B	82.7 (5)
O1W—Ni1A—Ni1B—O11A	30.3 (2)	N2—Ni1B—N1B—C3B	168.9 (5)
N1—Ni1A—Ni1B—O11A	108.89 (18)	Ni1A—Ni1B—N1B—C3B	−121.1 (4)
N1A—Ni1A—Ni1B—O1	−32.4 (2)	O1B—Ni1B—N1B—C2B	178.6 (3)
O1A—Ni1A—Ni1B—O1	79.00 (16)	O1—Ni1B—N1B—C2B	85.0 (3)
O11A—Ni1A—Ni1B—O1	151.73 (19)	O1M—Ni1B—N1B—C2B	−89.9 (3)
O1W—Ni1A—Ni1B—O1	−177.97 (19)	N2—Ni1B—N1B—C2B	−3.7 (3)
N1—Ni1A—Ni1B—O1	−99.37 (16)	Ni1A—Ni1B—N1B—C2B	66.3 (4)
N1A—Ni1A—Ni1B—O1M	145.2 (2)	Ni1A—O1—C1—C2	−123.0 (5)
O1A—Ni1A—Ni1B—O1M	−103.37 (16)	Ni1B—O1—C1—C2	125.7 (5)
O11A—Ni1A—Ni1B—O1M	−30.64 (18)	Ni1A—O1—C1—C6	58.6 (5)
O1—Ni1A—Ni1B—O1M	177.62 (18)	Ni1B—O1—C1—C6	−52.7 (5)
O1W—Ni1A—Ni1B—O1M	−0.34 (18)	O1—C1—C2—C3	−173.7 (5)
N1—Ni1A—Ni1B—O1M	78.25 (16)	C6—C1—C2—C3	4.7 (8)
N1A—Ni1A—Ni1B—N2	67.03 (19)	C1—C2—C3—C4	−0.9 (9)
O1A—Ni1A—Ni1B—N2	178.43 (15)	C2—C3—C4—C5	−3.3 (9)
O11A—Ni1A—Ni1B—N2	−108.83 (18)	C2—C3—C4—Br	176.7 (5)
O1—Ni1A—Ni1B—N2	99.43 (16)	C3—C4—C5—C6	3.2 (8)
O1W—Ni1A—Ni1B—N2	−78.53 (17)	Br—C4—C5—C6	−176.7 (4)
N1—Ni1A—Ni1B—N2	0.06 (15)	C4—C5—C6—C1	0.9 (8)
N1A—Ni1A—O1—C1	36.4 (3)	C4—C5—C6—C7	179.0 (5)
O1A—Ni1A—O1—C1	128.4 (3)	O1—C1—C6—C5	173.8 (5)
O11A—Ni1A—O1—C1	−140.7 (3)	C2—C1—C6—C5	−4.7 (7)
N1—Ni1A—O1—C1	−47.3 (3)	O1—C1—C6—C7	−4.4 (7)
Ni1B—Ni1A—O1—C1	−122.2 (4)	C2—C1—C6—C7	177.1 (5)
N1A—Ni1A—O1—Ni1B	158.64 (13)	C5—C6—C7—N2	−114.7 (5)
O1A—Ni1A—O1—Ni1B	−109.37 (13)	C1—C6—C7—N2	63.5 (6)
O11A—Ni1A—O1—Ni1B	−18.51 (12)	C5—C6—C7—N1	128.4 (5)
N1—Ni1A—O1—Ni1B	74.85 (14)	C1—C6—C7—N1	−53.3 (6)
O1B—Ni1B—O1—C1	−129.7 (3)	C9—N2—C7—C6	−175.4 (4)
N1B—Ni1B—O1—C1	−37.6 (3)	C1B—N2—C7—C6	66.0 (5)
O11A—Ni1B—O1—C1	142.6 (3)	Ni1B—N2—C7—C6	−50.5 (5)
O1M—Ni1B—O1—C1	113.9 (7)	C9—N2—C7—N1	−49.7 (4)
N2—Ni1B—O1—C1	47.0 (3)	C1B—N2—C7—N1	−168.4 (4)
Ni1A—Ni1B—O1—C1	124.1 (3)	Ni1B—N2—C7—N1	75.2 (3)
O1B—Ni1B—O1—Ni1A	106.20 (13)	C1A—N1—C7—C6	−69.7 (5)
N1B—Ni1B—O1—Ni1A	−161.72 (14)	C8—N1—C7—C6	169.6 (4)
O11A—Ni1B—O1—Ni1A	18.45 (12)	Ni1A—N1—C7—C6	47.1 (5)
O1M—Ni1B—O1—Ni1A	−10.2 (8)	C1A—N1—C7—N2	166.1 (4)
N2—Ni1B—O1—Ni1A	−77.09 (14)	C8—N1—C7—N2	45.4 (4)
N1A—Ni1A—O1A—C9A	−8.3 (4)	Ni1A—N1—C7—N2	−77.1 (4)
O11A—Ni1A—O1A—C9A	171.1 (4)	C1A—N1—C8—C9	−145.4 (4)
O1—Ni1A—O1A—C9A	−107.6 (4)	C7—N1—C8—C9	−23.7 (4)
O1W—Ni1A—O1A—C9A	80.9 (4)	Ni1A—N1—C8—C9	101.4 (4)
Ni1B—Ni1A—O1A—C9A	−149.6 (3)	C7—N2—C9—C8	34.7 (5)
N1B—Ni1B—O1B—C9B	11.5 (4)	C1B—N2—C9—C8	155.4 (4)
O11A—Ni1B—O1B—C9B	−168.1 (4)	Ni1B—N2—C9—C8	−88.7 (4)
O1—Ni1B—O1B—C9B	111.9 (4)	N1—C8—C9—N2	−6.9 (5)
O1M—Ni1B—O1B—C9B	−77.7 (4)	C7—N1—C1A—C2A	82.8 (5)
Ni1A—Ni1B—O1B—C9B	153.2 (4)	C8—N1—C1A—C2A	−161.8 (4)
O1A—Ni1A—O11A—C1AA	−62.1 (4)	Ni1A—N1—C1A—C2A	−42.2 (4)
O1—Ni1A—O11A—C1AA	−157.0 (4)	C3A—N1A—C2A—C1A	145.2 (5)
O1W—Ni1A—O11A—C1AA	27.6 (4)	Ni1A—N1A—C2A—C1A	−27.4 (5)
N1—Ni1A—O11A—C1AA	116.2 (4)	N1—C1A—C2A—N1A	48.7 (6)
Ni1B—Ni1A—O11A—C1AA	−176.0 (5)	C2A—N1A—C3A—C4A	−179.3 (5)
O1A—Ni1A—O11A—Ni1B	113.87 (14)	Ni1A—N1A—C3A—C4A	−7.8 (7)
O1—Ni1A—O11A—Ni1B	18.99 (13)	N1A—C3A—C4A—C5A	−176.9 (5)
O1W—Ni1A—O11A—Ni1B	−156.43 (15)	N1A—C3A—C4A—C9A	0.5 (8)
N1—Ni1A—O11A—Ni1B	−67.78 (15)	C9A—C4A—C5A—C6A	1.0 (7)
O1B—Ni1B—O11A—C1AA	64.0 (4)	C3A—C4A—C5A—C6A	178.5 (5)
O1—Ni1B—O11A—C1AA	157.6 (4)	C4A—C5A—C6A—C7A	1.1 (8)
O1M—Ni1B—O11A—C1AA	−27.4 (4)	C4A—C5A—C6A—Br1A	−176.8 (4)
N2—Ni1B—O11A—C1AA	−113.7 (4)	C5A—C6A—C7A—C8A	−1.0 (8)
Ni1A—Ni1B—O11A—C1AA	176.4 (5)	Br1A—C6A—C7A—C8A	176.9 (4)
O1B—Ni1B—O11A—Ni1A	−112.37 (15)	C6A—C7A—C8A—C9A	−1.3 (8)
O1—Ni1B—O11A—Ni1A	−18.76 (13)	Ni1A—O1A—C9A—C4A	4.7 (7)
O1M—Ni1B—O11A—Ni1A	156.15 (14)	Ni1A—O1A—C9A—C8A	−173.8 (3)
N2—Ni1B—O11A—Ni1A	69.94 (15)	C5A—C4A—C9A—O1A	178.4 (4)
O1B—Ni1B—O1M—C1M	39.2 (4)	C3A—C4A—C9A—O1A	1.1 (8)
N1B—Ni1B—O1M—C1M	−52.3 (4)	C5A—C4A—C9A—C8A	−3.1 (7)
O11A—Ni1B—O1M—C1M	127.5 (4)	C3A—C4A—C9A—C8A	179.6 (5)
O1—Ni1B—O1M—C1M	155.8 (6)	C7A—C8A—C9A—O1A	−178.1 (5)
N2—Ni1B—O1M—C1M	−137.1 (4)	C7A—C8A—C9A—C4A	3.3 (7)
Ni1A—Ni1B—O1M—C1M	147.2 (4)	C9—N2—C1B—C2B	165.2 (4)
N1A—Ni1A—N1—C1A	20.9 (3)	C7—N2—C1B—C2B	−81.6 (5)
O11A—Ni1A—N1—C1A	−158.6 (3)	Ni1B—N2—C1B—C2B	41.2 (4)
O1—Ni1A—N1—C1A	120.5 (3)	C3B—N1B—C2B—C1B	−146.4 (5)
O1W—Ni1A—N1—C1A	−68.4 (3)	Ni1B—N1B—C2B—C1B	26.8 (5)
Ni1B—Ni1A—N1—C1A	162.6 (3)	N2—C1B—C2B—N1B	−46.9 (6)
N1A—Ni1A—N1—C7	−102.2 (3)	C2B—N1B—C3B—C4B	176.4 (5)
O11A—Ni1A—N1—C7	78.3 (3)	Ni1B—N1B—C3B—C4B	4.2 (8)
O1—Ni1A—N1—C7	−2.7 (3)	N1B—C3B—C4B—C5B	−176.6 (5)
O1W—Ni1A—N1—C7	168.4 (3)	N1B—C3B—C4B—C9B	1.8 (8)
Ni1B—Ni1A—N1—C7	39.4 (3)	C9B—C4B—C5B—C6B	1.1 (7)
N1A—Ni1A—N1—C8	140.3 (3)	C3B—C4B—C5B—C6B	179.5 (5)
O11A—Ni1A—N1—C8	−39.2 (3)	C4B—C5B—C6B—C7B	−0.9 (8)
O1—Ni1A—N1—C8	−120.1 (3)	C4B—C5B—C6B—Br1B	−179.4 (4)
O1W—Ni1A—N1—C8	51.0 (3)	C5B—C6B—C7B—C8B	0.7 (8)
Ni1B—Ni1A—N1—C8	−78.0 (3)	Br1B—C6B—C7B—C8B	179.3 (4)
N1B—Ni1B—N2—C9	−140.4 (3)	C6B—C7B—C8B—C9B	−0.7 (8)
O11A—Ni1B—N2—C9	39.2 (3)	Ni1B—O1B—C9B—C4B	−9.8 (7)
O1—Ni1B—N2—C9	119.3 (3)	Ni1B—O1B—C9B—C8B	170.7 (3)
O1M—Ni1B—N2—C9	−50.9 (3)	C5B—C4B—C9B—O1B	179.5 (5)
Ni1A—Ni1B—N2—C9	78.0 (3)	C3B—C4B—C9B—O1B	1.2 (7)
N1B—Ni1B—N2—C7	102.8 (3)	C5B—C4B—C9B—C8B	−1.0 (7)
O11A—Ni1B—N2—C7	−77.5 (3)	C3B—C4B—C9B—C8B	−179.3 (5)
O1—Ni1B—N2—C7	2.5 (3)	C7B—C8B—C9B—O1B	−179.6 (5)
O1M—Ni1B—N2—C7	−167.7 (3)	C7B—C8B—C9B—C4B	0.8 (7)
Ni1A—Ni1B—N2—C7	−38.8 (3)	Ni1A—O11A—C1AA—O2AA	−154.8 (4)
N1B—Ni1B—N2—C1B	−20.0 (3)	Ni1B—O11A—C1AA—O2AA	30.2 (7)
O11A—Ni1B—N2—C1B	159.6 (3)	Ni1A—O11A—C1AA—C2AA	25.4 (7)
O1—Ni1B—N2—C1B	−120.3 (3)	Ni1B—O11A—C1AA—C2AA	−149.6 (4)

### Hydrogen-bond geometry (Å, °)

**Table:**

*D*—H···*A*	*D*—H	H···*A*	*D*···*A*	*D*—H···*A*
O1W—H1W1···O2W^i^	0.80 (2)	2.01 (2)	2.810 (5)	174 (5)
O1W—H1W2···O3M	0.82 (2)	1.97 (3)	2.770 (5)	164 (5)
O2W—H2W1···O1B	0.80 (2)	1.86 (3)	2.631 (5)	160 (6)
O2W—H2W2···O1A	0.83 (2)	1.91 (2)	2.744 (5)	177 (6)
O1M—H1M···O2AA	0.84	1.77	2.602 (5)	174.
O2M—H2M···O2W	0.84	1.89	2.725 (5)	172.
O3M—H3M···O2M^i^	0.84	1.96	2.753 (6)	158.

Symmetry codes: (i) *x*−1/2, −*y*+3/2, *z*.
